# Diversity-Oriented Synthetic Strategies Applied to Cancer Chemical Biology and Drug Discovery

**DOI:** 10.3390/molecules191117221

**Published:** 2014-10-27

**Authors:** Ian Collins, Alan M. Jones

**Affiliations:** 1 Cancer Research UK Cancer Therapeutics Unit, The Institute of Cancer Research, London SM2 5NG, UK; 2 Division of Chemistry and Environmental Science, School of Science and the Environment, Faculty of Science and Engineering, Manchester Metropolitan University, John Dalton Building, Chester Street, Manchester M1 5GD, UK

**Keywords:** diversity-oriented synthesis (DOS), biology-oriented synthesis (BIOS), cancer, chemical tools, drug discovery

## Abstract

How can diversity-oriented strategies for chemical synthesis provide chemical tools to help shape our understanding of complex cancer pathways and progress anti-cancer drug discovery efforts? This review (surveying the literature from 2003 to the present) considers the applications of diversity-oriented synthesis (DOS), biology-oriented synthesis (BIOS) and associated strategies to cancer biology and drug discovery, summarising the syntheses of novel and often highly complex scaffolds from pluripotent or synthetically versatile building blocks. We highlight the role of diversity-oriented synthetic strategies in producing new chemical tools to interrogate cancer biology pathways through the assembly of relevant libraries and their application to phenotypic and biochemical screens. The use of diversity-oriented strategies to explore structure-activity relationships in more advanced drug discovery projects is discussed. We show how considering appropriate and variable focus in library design has provided a spectrum of DOS approaches relevant at all stages in anti-cancer drug discovery.

## 1. Introduction

Diversity-oriented synthetic (DOS) approaches can provide new chemical tools to help shape our understanding of complex cancer biology, and a platform for the identification and progression of anti-cancer drug leads. The DOS approach is undergoing resurgence in terms of its application to medicinal chemistry due to the architecturally complex products that result. These can have high non-aromatic carbon (fraction of sp^3^ hybrid atoms; Fsp^3^) and chiral content, which have been less common features of hit generation libraries to date [1]. More diverse and complex scaffolds are seen as one way to extend the reach of drug discovery to biomolecular interactions that have been viewed so far as less tractable for small molecule modulation, such as highly conformationally flexible proteins, protein-protein interactions [2], and protein-nucleic acid recognition sites.

**Figure 1:**
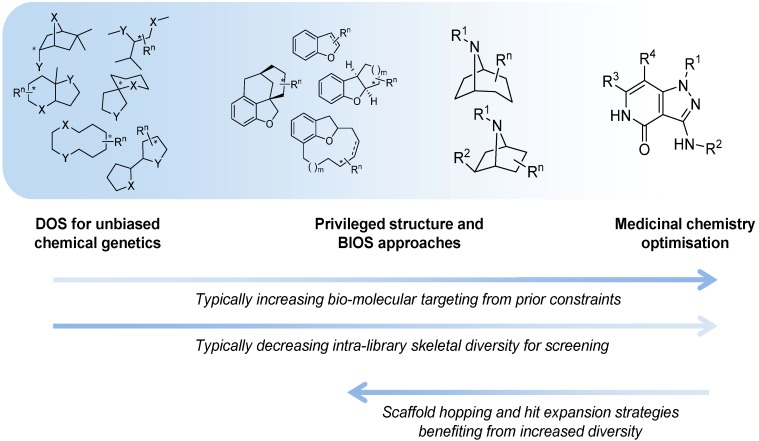
The spectrum of diversity-oriented approaches applied to make, find and improve biologically active small molecule chemical tools and drug leads.

One of the original aims of DOS was to populate undeveloped chemical space using inventive yet simple to perform reactions to generate novel chemical scaffolds. This would allow the exploration of new areas of structural space to discover new biologically active molecules primarily as tools for chemical genetics, but also to provide a new chemical pool for drug discovery [3]. However, DOS does not exist in isolation to other strategies for making, identifying and refining biologically active small molecules. Other approaches to discovering biological active small molecules such as: virtual screening [4,5]; pharmacophore modelling [6]; fragment-based approaches [7]; and network-based approaches [8] are also important in both the early discovery phase and later optimisation of lead compounds. However, cross-fertilisation between DOS, the above approaches and other more established approaches has been fruitful, and it is possible to view the current position as a spectrum of design strategies that varies by the prominence given to skeletal structural diversity [9,10,11,12,13,14] (Figure 1).

At one extreme DOS may aim to maximise the structural diversity of the compounds made and to populate structural space or achieve complexity that has been under-represented in conventional libraries. The availability of compounds with new structural types and greater complexity may be especially relevant for tackling molecular targets or interactions that have so far proved resistant or difficult to find small molecule modulators for, in currently explored chemical space. In anti-cancer drug discovery, specific modulators of protein-protein or protein-nucleic acid interactions might be considered in this context. Unbiased approaches to DOS library design that maximise diversity and complexity have the disadvantage that the likelihood of encountering bioactivity with the compound library in any given screen is unknown and possibly very low, and may require very large libraries to mitigate this. 

On the other hand it is possible to consider constraints to the diversity during the forward synthesis design stage to provide a varying level of focus to the DOS libraries, with the aim of tailoring them to increase the likelihood of hits in specific screening settings or against selected molecular targets. A potential disadvantage of applying existing knowledge is to gravitate towards what has been explored previously, and potentially to restrict the possibility of new findings or of overturning unhelpful dogma, compared to an unbiased approach.

When considering DOS applied to drug discovery, the ultimate need is to find bioactive compounds compatible with the environment of the human body that are stable to metabolising enzymes and balance the conflicting physicochemical demands of water solubility and lipid membrane permeability required for a drug molecule to penetrate tissues and cells. Thus the physicochemical property ranges associated with bioavailability, regardless of biological target, are useful constraints to apply to DOS library design in the context of medicinal chemistry and drug discovery. Likewise, physicochemical properties or functional groups associated with higher probability of organism-level toxicity could usefully be avoided [14,15].

Similarity to known bioactive molecules, such as endogenous metabolites, natural products, xenobiotic drug molecules or chemical tools, is one way to select scaffolds for more focussed DOS approaches to tackling specific targets or mechanisms. Thus, Biology-Oriented Synthesis (BIOS) as proposed by Waldmann and colleagues [12,13] identifies promising groups of scaffolds for DOS elaboration from analysis of the scaffolds of known bioactive compounds. The selection can be of varied stringency and applied at different levels in the scaffold analysis, and is therefore compatible with the idea of chemical-diversity driven DOS [16]. At the extreme of the spectrum of limitations on skeletal diversity that may be considered, this approach becomes analogous to the privileged structure strategy familiar to medicinal chemists [17,18] which typically involves substituent decoration of single scaffolds selected for their propensity to show particular bioactivities. In this context, it is interesting to note that the most obvious perceived chemical structural diversity may not always coincide with diverse biological behaviour, and that skeletal, substituent and stereochemical diversity must all be considered. At the highest structural resolutions available to molecular engineers in organic chemistry—the addition, removal or replacement of a single atom or the inversion of a single stereocentre—minimal changes may result in quite profound changes in biological activity despite high apparent similarity in the structural framework. Likewise, apparently structurally diverse compounds may converge on the same bioactivities [17,19]. The techniques of DOS may also be applied to expand scaffold diversity from single starting points within medicinal chemistry projects. This could be of particular importance when an initial hit lacks novelty or target specificity, or in structure-based drug discovery when the first scaffold discovered lacks synthetically tractable vectors to explore regions of interest in the binding site.

The organisation of material in this review will follow the broad pattern in Figure 1; starting with chemical probe discovery from DOS libraries put through screens directly tackling phenotypes or classes of interactions relevant to cancer. We will then survey the applications of DOS to anti-cancer drug discovery where a broad initial approach is taken, such as to construct a diverse screening library. Finally, we will discuss more tightly targeted drug discovery, where a single start point with specific activity is expanded and explored through DOS.

## 2. DOS as a Source of New Chemical Tools for Cancer Biology

**Figure 2:**
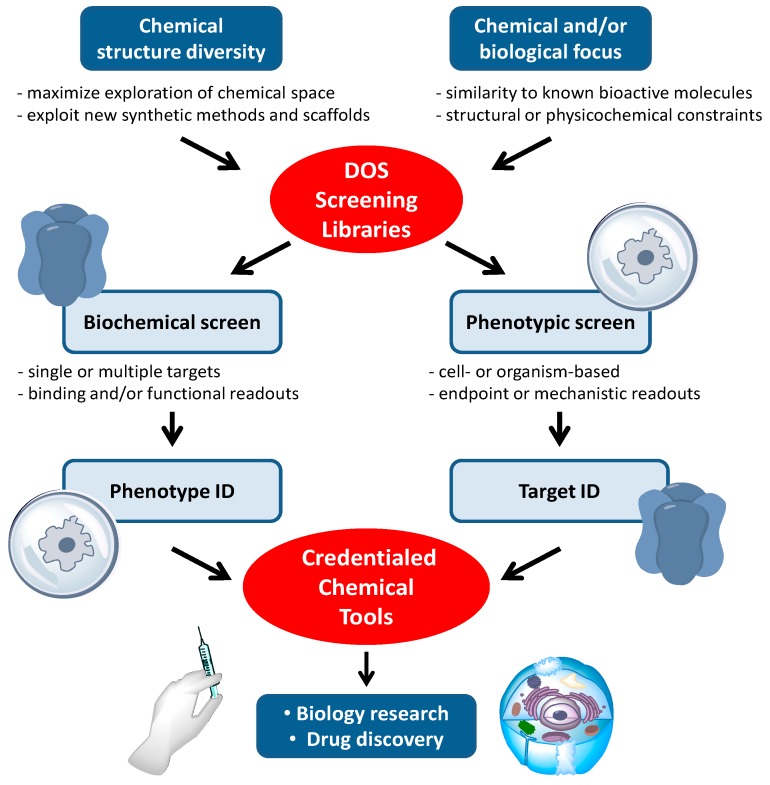
The application of DOS screening libraries to the discovery of new chemical tool compounds for cancer biology research and anticancer drug discovery.

Compound libraries generated by DOS are well established sources of new, biologically active chemical tools [9]. An overview of the typical work-flows for the application of DOS libraries to cancer biology research and drug discovery is shown in Figure 2. Many, but by no means all, of the biological attributes of cancer can be observed in cells grown in culture. *In vitro* phenotypic screens are readily conducted with human cancer cell lines, and this may be a factor in the observed high frequency with which DOS approaches have been applied to tackle cancer biology questions in the past decade. Phenotypic screens in cancer have often been focussed on general and potentially non-specific endpoints, such as cytotoxicity or anti-proliferative effects, which can limit interpretation of the mode of action of hit compounds [20]. Increasingly, the screening endpoints are related to more specific changes in cell morphology, behaviours such as motility and invasiveness, or to informative mechanistic readouts, e.g., arrest in specific phases of the cell cycle or evidence of apoptotic cell death. Pathway-driven reporter gene assays are widely used to identify inhibitors targeting specific signalling pathways in the cell, while synthetic lethal screens which combine RNA interference with small molecules in the cancer cell, can inform on the effects of modulating combinations of targets. Single cell imaging allows changes in the cell architecture to be probed, e.g., mitotic spindle disruption, or single molecules to be tracked, e.g., by following intracellular re-localisation of labelled proteins. Medium-to-high throughput phenotypic screening at the small organism level extends the range of potential phenotypes that can be seen and modulated by small molecules. For example angiogenesis, a key hallmark of cancer is readily observed in zebra-fish embryos.

The contemporary approach to small molecule cancer drug discovery aims to intervene in the function of specific molecular targets, usually proteins, based on an understanding of the genetic and epigenetic changes that lead to cancer. These molecular targets may be mutated oncogenic proteins with a gain of function that drives malignancy, proteins that are dis-regulated as a result of oncogenic changes elsewhere in signalling networks, or epigenetic modifiers of gene product expression [21,22]. Additionally, rapidly proliferating cancer cells co-opt a number of non-oncogenic stress response pathways in order to survive, for example to mitigate DNA damage due to genomic instability or to chaperone and stabilise poorly folded oncogenic proteins [21,23]. Thus, as well as identifying and characterising desirable phenotypic changes induced by small molecule tool compounds, an understanding of the precise molecular targets of the compounds is required. The order in which these activities occurs can vary but both phenotypic and target functional assays are needed to derive well characterised chemical tools for cancer biology research from DOS approaches.

**Figure 3:**
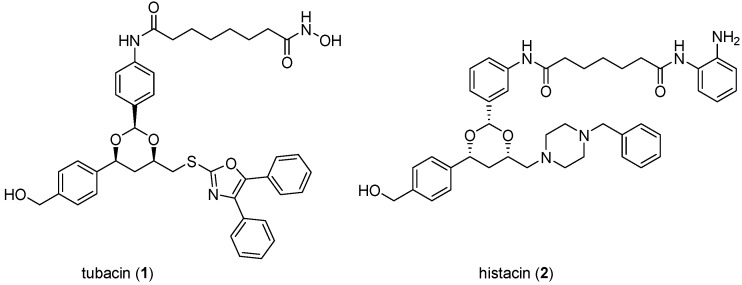
Structures of tubacin and histacin HDAC inhibitors.

**Figure 4:**
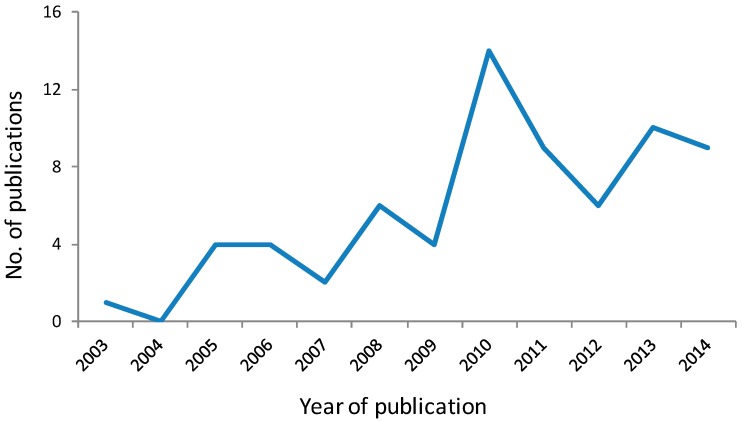
Primary journal reports identified from the Chemical Abstracts database as citing the use of tubacin as a chemical tool in biological studies. Data for 2014 is for the first 6 months [26].

The importance to cancer biology research of chemical tools discovered through DOS is illustrated by tubacin (**1**; Figure 3), one of the earliest reported molecules to be identified through phenotypic screening of an explicit DOS library [24,25]. In the decade since its discovery, this selective histone deacetylase (HDAC) 6 inhibitor has been widely and increasingly used in biological studies to elucidate the role of HDAC6 and tubulin acetylation in the biology of cancer and other diseases, as shown by a recent citation search of the Chemical Abstracts database where approximately 100 primary publications were identified using tubacin as a chemical tool (Figure 4).

In the discovery of tubacin a library of 7392 1,3-benzodioxanes was constructed by DOS on solid support where the library components were biased towards deacetylase inhibition through the inclusion of terminal zinc-binding motifs (*ortho*-anilide, hydroxamic acid, carboxylic acid) attached to long chain spacers similar to those found in the prototypic natural product HDAC inhibitors trichostatin A and trapoxin [27]. The library was screened in parallel in two cell-based assays to identify molecules that caused selective inhibition of either nuclear or cytoplasmic deacetylase function, monitored by histone and α-tubulin acetylation, respectively [24]. Tubacin (**1**) was identified as the first known selective inhibitor of α-tubulin deacetylation, while histacin (**2**, Figure 3) was found to be a selective inhibitor of histone deacetylation. Subsequent analysis of tubacin’s mechanism of action showed that the compound inhibited the class II HDAC6 isoform and reduced cell motility and migration [25].

**Scheme 1:**
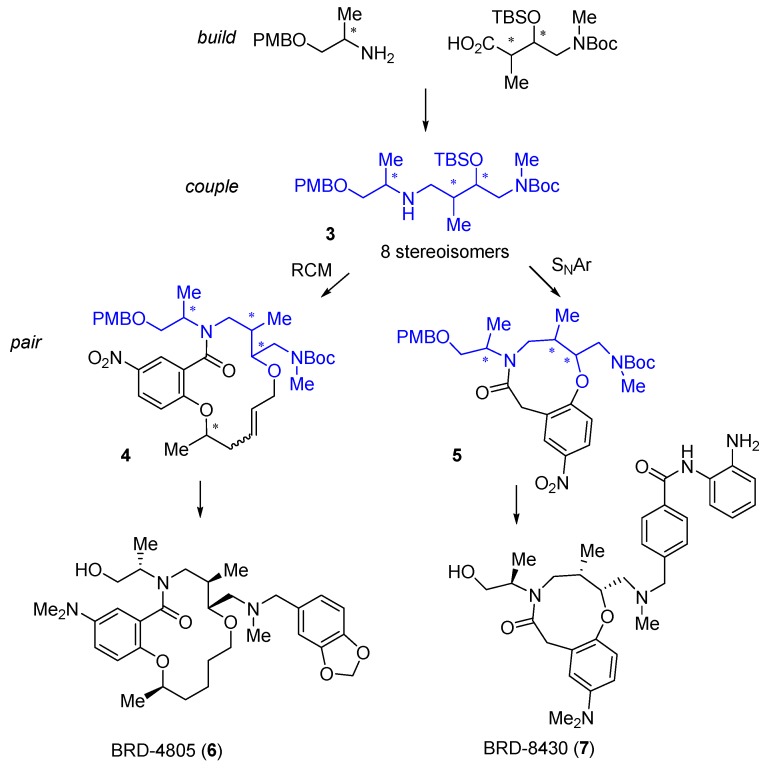
DOS of macrocyclic HDAC2 inhibitors and the structures of isoform selective HDAC inhibitors.

A very different DOS library based on scaffold diversity was applied to the discovery of novel macrocyclic HDAC inhibitors [28]. A *build-couple-pair* synthetic strategy was used to construct all 8 stereoisomers of the linear amine **3** (Scheme 1) followed by ring-closing metathesis (RCM) with the introduction of a fourth stereocentre to construct 16 stereoisomers of the 14-membered macrocycle **4**. The RCM in the intramolecular cyclisation could be replaced by intramolecular S_N_Ar or 1,3-dipolar cycloaddition reactions to generate sets of 8-, 9- (e.g., **5**), 12- or 13-membered cyclic scaffolds. A library of 14,400 compounds based on substituent diversification was prepared on solid phase starting from the RCM scaffolds **4**. The compound sets were screened in multiple biochemical and cell-based assays, including an HDAC2 biochemical screen in which a total of 22,506 compounds were tested, including 1604 14-membered lactams. Follow-up structure-activity studies around interesting RCM macrolactam hits identified the stereochemically pure benzo-1,2-dioxolane BRD-4805 (**6**) as a micro-molar inhibitor of HDAC1-3, with selectivity against HDAC4-8, that increased histone acetylation in mouse neuronal cells. Importantly, the compound lacked the characteristic zinc-chelating functionality usually associated with HDAC inhibitors, and showed an unusual mixed competitive mode of inhibition. Incorporation of terminal *ortho*-anilide substitution into the library design was used to bias the library towards class I HDAC inhibition, resulting in the identification of the 9-membered lactam BRD-8430 (**7**) as a selective inhibitor of HDAC1 (IC_50_ 69 nM) [29] with some activity against HDAC2 and three isoforms. Using BRD-8430 and other HDAC inhibitors with different selectivity profiles, in parallel with combinatorial RNAi studies, the authors showed that dual inhibition of HDAC1 and 2 decreased viability and induced differentiation of neuroblastoma cell lines. The biology of isoform-selective inhibition of HDACs is a topic of current interest in cancer research [30], to which DOS-derived ligands are making substantial contributions.

**Scheme 2:**
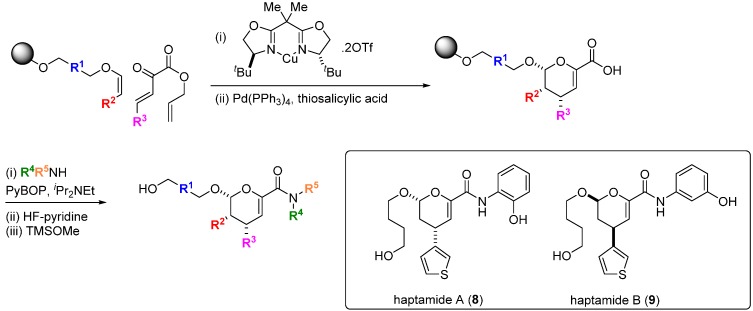
DOS of dihydropyrancarboxamides and structures of inhibitors of HAP-family transcription factors.

Transcription factors are key regulators of oncogenic gene programs but direct and specific modulation of the interactions of these proteins with their DNA binding sites or with other protein co-regulators has proved generally difficult to achieve. It might be expected that the more structurally complex DOS libraries would provide greater opportunities to find small molecules capable of disrupting these interactions [31]. An early example of this was provided by the discovery of the small molecule haptamides A and B (**8**, **9**; Scheme 2) as direct binders and inhibitors of the yeast transcription factor Hap3p [32]. Using a one bead-one stock solution format, a 12,396 compound microarray was assembled using three alternative DOS pathways. The library contained amongst others, examples of dihydropyrancarboxamides, 1,3-dioxanes and biaryl-containing medium rings. The microarrays were probed with Hap3p-GST fusion protein and binding was detected with a Cy5-labelled antibody against the GST component of the fusion protein, from which haptamide A (**8**) was identified as the sole specific hit (SPR K_d_ 5 µM). Haptamide A reversibly inhibited expression of a HAP3p-dependent reporter gene in yeast cells (IC_50_ 42 µM). Rapid exploration of the structure-activity relationships (SAR) around **8** was possible using the DOS route with solid phase synthesis on alkylsilyl macrobeads. A stereospecific hetero-Diels-Alder reaction catalysed by a Cu-bisoxazoline (BOX) ligand system installed the core scaffold, followed by palladium-catalysed allyl group deprotection, amide bond coupling and fluoride-mediated silyl polymer support deprotection, leading to haptamide B (**9**) with improved potency (K_d_ 330 nM; IC_50_ 24 µM). Although the mechanism of inhibition was not determined, whole-genome transcription profiling showed that **9** selectively inhibited transcription regulated by the family of Hap2/3/4/5p transcription factors.

**Figure 5:**
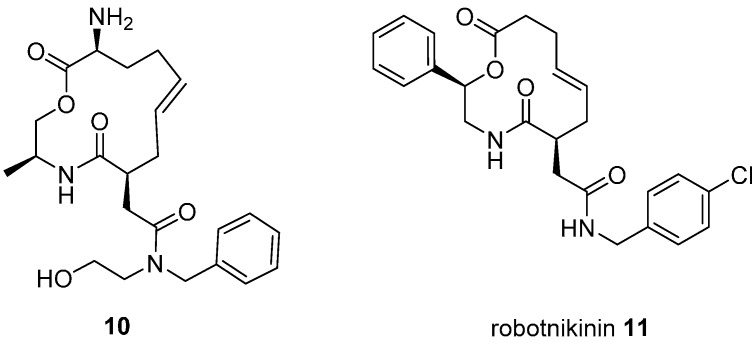
Structures of macrocyclic inhibitors of sonic Hedgehog extracellular signalling protein.

An alternative strategy for modulating transcription is to target signalling pathways upstream of the transcription factors themselves. This was demonstrated for macrocyclic inhibitors of the extracellular protein sonic hedgehog (Shh) which regulates downstream activation of the Gli family of transcription factors, important in embryo development and some cancers [33]. Although inhibitors of Shh signalling had previously been identified in phenotypic screens, none were known to target the protein directly. A DOS library of 10,000 compounds was screened for binding to recombinant Shh leading to the 13-membered macrocyclic ligand **10** (Figure 5; K_d_ 9 µM) which inhibited Shh-activated, Gli transcription factor-dependent reporter gene expression in cultured fibroblasts. Structure-activity studies identified the 12-membered analogue robotnikinin (**11**; K_d_ 3.1 µM) with increased dissociation time from Shh. Studies in engineered and primary human skin cells suggested that robotnikinin acted by inhibiting formation of the complex between Shh and its extracellular binding partners with its transmembrane receptor, Patched.

The Wnt proteins are another group of extracellular proteins that initiate signalling cascades that can lead to transcriptional activation and are important in carcinogenesis and cell differentiation. A small BIOS-derived library of 91 substituted oxepanes and oxepane-containing frameworks was designed, based on recognition of the oxepane ring as a privileged scaffold embedded in diverse natural products with varying biological activity [34].

**Scheme 3:**
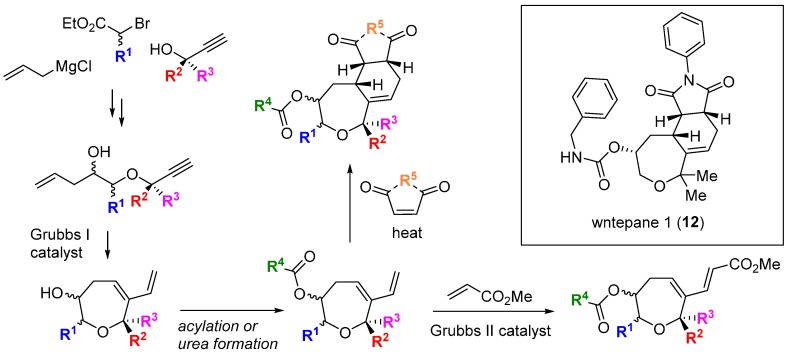
BIOS and structure of an activator of Wnt signalling.

Diels-Alder cycloadditions to intermediate 6-vinyl-2,3,4,7-tetrahydrooxepines were used to diversify the core scaffold. The compounds were screened in a cell reporter assay sensitized to detect stimulation of Wnt3a-dependent transcription. Pathway activators that acted synergistically with the presence of the Wnt3a isoform were detected, and subsequent SAR studies identified the activator wntepane 1 (**12**; Scheme 3; ED_50_ 1.8 µM). An affinity probe based on **12** linked to biotin was used to capture potential targets and reversible binding to the Wnt membrane receptor Vangl1 was demonstrated, the first time a tool molecule targeting this protein was reported.

**Scheme 4:**
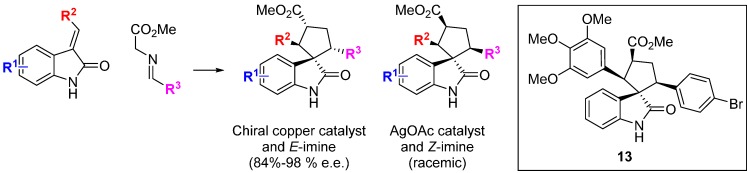
BIOS of spirooxindole inhibitors of microtubule organization.

The rearrangement of cell architecture during mitosis lends itself to high-content phenotypic screens, as shown by the identification of the spirooxindole **13** (Scheme 4) [35]. The starting point for this BIOS approach was the recognition of the spirooxindole motif in many bioactive compounds, including the tubulin polymerization inhibitor spirotryprostatin B. A highly enantioselective, copper-catalyzed 1,3-dipolar cycloaddition of glycine ester *E*-imines and benzylidene- or alkylidene-oxindoles was developed to construct a small library of 3,3'-pyrrolidinyl spirooxindoles. The general use of catalytic enantioselective 1,3-dipolar cycloadditions of azomethine ylides in BIOS has been highlighted and reviewed [36]. Changing the Lewis-acid catalyst to silver acetate and using a stereochemically pure *Z*-imine starting material gave racemic products with a different stereochemistry in the pyrrolidine ring. The analogue **13** was identified from a screen of 39 compounds as the only hit that caused mitotic arrest, evidenced by changes in cell morphology and DNA content as well as effects on proliferation. Despite the scaffold similarity to known spirooxindole inhibitors of p53-MDM2, **13** was not an inhibitor of this protein-protein interaction. Rather, **13** was found to interfere with microtubule organization and nucleation centre distribution, but without the direct effects on microtubule polymerization seen with spirotryprostatin B. Consistent with this, in HeLa human cancer cells **13** caused multipolar spindle formation, lagging chromosomal separation and mitotic arrest. This example nicely illustrates some of the strengths and potential challenges of applying diversity-oriented approaches to chemical probe identification. Firstly, the BIOS logic focusses the chemical space explored to efficiently identify new compounds with bioactivity in cells, and the 1/39 hit rate (2.6%) contrasts with other examples discussed above where libraries of several thousands of more structurally diverse compounds were screened to identify hits. However, despite high scaffold similarity to known bioactive compounds the spirooxindole **13** has a cellular phenotypic profile that would not necessarily be predicted from the biology of the known compounds. As the precise molecular target(s) of the compound are not automatically recovered from the phenotypic screen, further work would be required to credential the molecule for use as a chemical tool, but this example also clearly shows the advantage of high-content phenotypic screens in providing in-depth characterisation and differentiation of the hit molecules.

**Scheme 5:**
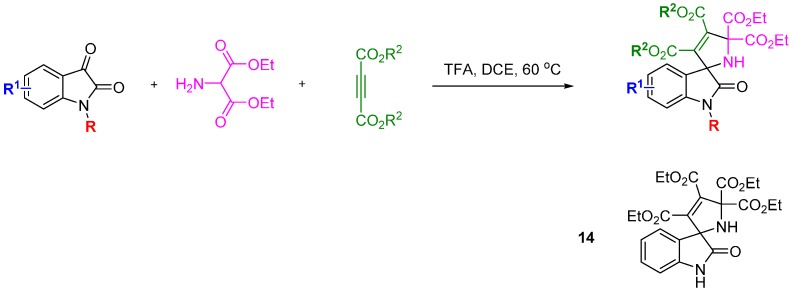
A three-component reaction to a 2,5-dihydropyrrole spirooxindole-based library.

The privileged spirooxindole ring system was used as inspiration for a DOS library by the research groups of Zhang and Shi [37]. The 1,3-dipolar cycloaddition reaction between a range of electron deficient alkynes and isatin-derived azomethine ylides afforded 36 spirooxindole-based 2,5-dihydropyrrole compounds in good to excellent yield (Scheme 5). Several compounds exhibited modest cytotoxic effects *in vitro* in MCF-7 cells (up to 28% inhibition at 100 µg/mL). The possible mechanism of the cytotoxicity of this compound was investigated using Hoechst 33 324 staining to determine whether the cytotoxic effects were related to cell apoptosis. A dose-dependent response was found by flow cytometry studies indicating that the observed cytotoxicity was due to cell apoptosis. To elucidate the mechanism of apoptosis the levels of the signalling kinases ERK1/2, p38 and JNK and their respective phosphorylation states was measured. Levels of p38 and pJNK increased after 30 minutes, followed by pERK1/2 after 1 h, and remained high for 8 h post treatment. Separate pre-treatment of MCF-7 cells with a MEK1, ERK1/2 or JNK inhibitor, respectively, did not affect the apoptotic response and it was concluded that compound **14** acted on MCF-7 cells through the MAPK pathway.

**Scheme 6:**
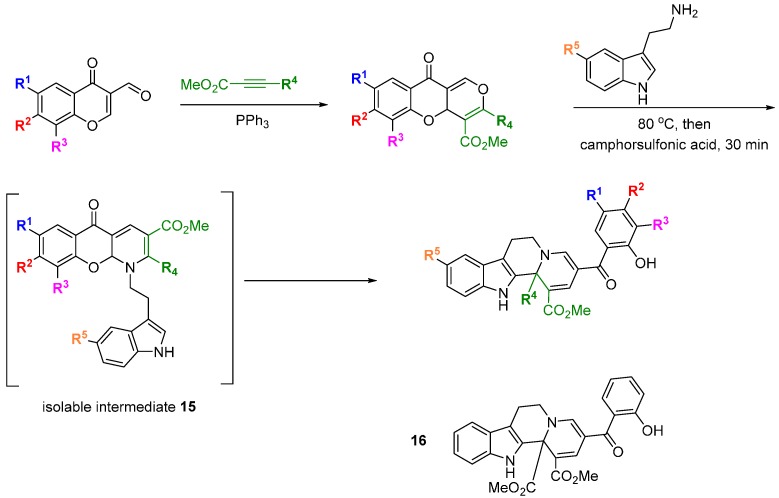
Cascade synthesis of a BIOS library of indoloquinolizines.

Waldmann and Kumar used a natural product-inspired synthetic cascade to prepare a BIOS library and identified modulators of centrosome integrity [38]. An elegant cascade sequence of twelve steps was used to prepare tetracyclic indoloquinolizines resembling the structure of polycyclic indole alkaloids (Scheme 6). Electron deficient 3-formyl chromones and alkynes treated with triphenylphosphine, acting as a *pseudo* organocatalyst, underwent a [4 + 2] annulation to afford a range of benzopyrones. Reaction of the benzopyrones with tryptamines resulted in aminals via a regioselective S_N_2'-type addition that opened the chromone ring and liberated the phenol. The aminals underwent conjugate re-addition of the phenol, followed by S_N_2'-type retro-Michael reaction and pyran ring opening to generate an enamine, which in turn cyclised on to the proximal carbonyl group to install a dihydropyridine ring. The *para* relationship between the dihydropyridine nitrogen and chromone oxygen led to a second chromone ring-opening and regenerated the phenolic group. Rotation around the acyl-pyridinium C-C bond allowed the newly generated phenol to intercept the pyridinium ring at the C2 position, generating intermediates **15** that could be isolated. Aza-Claisen rearrangement and subsequent ring-opening revealed a system with the substituents arranged to undergo a Pictet-Spengler cyclisation. The tetrahydrocarboline generated, underwent an Aza-Michael addition followed by retro-Michael addition and another chromone ring-opening to afford the final indoloquinolizine products. The focussed collection was screened for modulation of cell division using a phenotypic screen in BSC-1 cells. Compound **16** led to the accumulation of rounded cells with condensed DNA, a feature of mitotic cells, and also had the effect of inducing three daughter cells during mitosis in HeLa and U2OS cells overexpressing tubulin. The SAR generated from the initial 26 indoloquinolizines suggested groups pendant from the R^5^-position would not be detrimental to activity and allowed a linker to be placed to immobilize the active compound to Sepharose beads. Pull down experiments with this affinity probe (compared with a negative control) in HeLa cells lysates led to the conclusion that **16** reversibly bound to the centrosomal protein NPM (and Crm1) and U2 small ribonuclear protein. The affinities (K_d_) for binding to His-NPM and Crm1 were determined using a fluorescent probe (a derivative of **16**, not shown) as 25 and 9 µM, respectively. HeLa cells treated with **16** at 50 µM demonstrated partial inhibition of nuclear export, consistent with Crm1 targeting. Cytotoxicity assays showed **16** to be significantly less toxic than leptomycin B, a known nuclear export inhibitor. Thus the application of a BIOS approach led to a novel inhibitor of centrosome-associated proteins NPM and Crm1 in cells and further analysis revealed the effects of **16** on centrosome and spindle integrity, chromosome defects, M stage cell cycle arrest and ultimately, apoptosis.

**Scheme 7:**
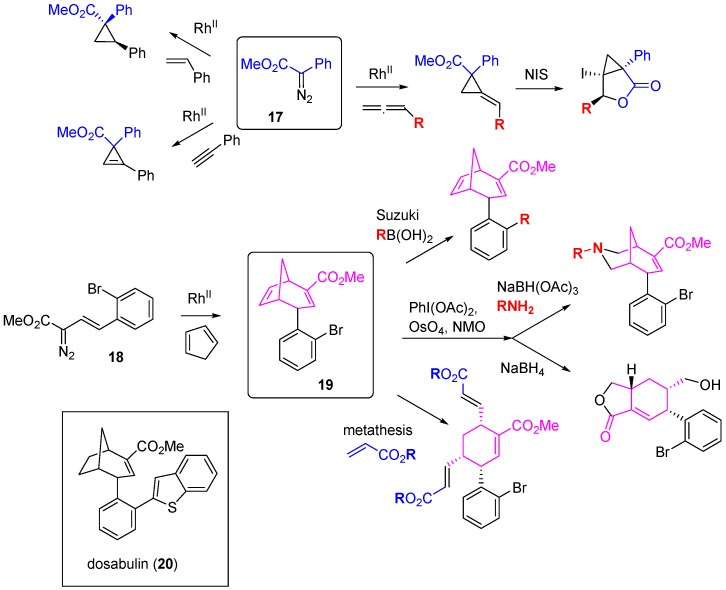
Rhodium carbenoid methodology used for DOS leading to identification of inhibitors of tubulin polymerization.

A high-content phenotypic screen for antimitotic activity was also applied to a structurally diverse DOS library constructed using rhodium-catalyzed carbene formation and insertion chemistry [39]. Phenyl diazoester **17** was the starting point for the synthesis of a sub-library of scaffolds through intermolecular cyclopropanation of alkenes, alkynes and allenes (Scheme 7). The styryl diazoester **18** underwent tandem cyclopropanation-Cope rearrangement with cyclopentadiene to give the bicyclo[3.2.1]octadiene **19**. In turn, divergent reactions of **19** through Suzuki couplings, alkene epoxidation or hydroxylation, metathesis and oxidative alkene cleavage generated a library of 35 compounds covering 10 distinct scaffolds. Chemoinformatic analysis demonstrated a significant shift in the DOS library towards more spherical shapes (as defined by principal moments of inertia) compared to a collection of known drugs. In U2OS human osteosarcoma cells, two bicyclo[3.2.1]octadienes caused mitotic arrest and follow up synthesis of reduced analogues identified the *S*-enantiomer of dosabulin (**20**) as a more potent inhibitor of mitosis (EC_50_ 1.2 µM) that caused cell death through apoptosis. Confocal microscopy showed disruption of the tubulin network in cells treated with (*S*)-**20** and the compound was an inhibitor of tubulin polymerization *in vitro*. Competition studies indicated that (*S*)-**20** was not binding to the vinblastine site on tubulin, but was binding near or in allosteric relation to the colchicine binding site.

One strategy to enable easier deconvolution of the molecular targets of a probe compound identified in phenotypic screening is to use the concept of synthetic lethality in the design of the cellular screening approach. Here a specific genetic modification or chemical inhibitor is introduced that, although itself inadequate to perturb the phenotype, renders the cells sensitive to additional inhibition of compensatory pathways.

**Scheme 8:**
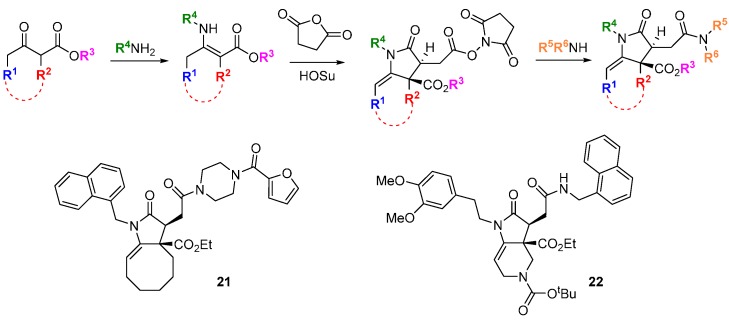
Identification of inhibitors of glucose uptake from a DOS library of pyrrolidinones.

Screening is carried out in the absence and presence of the specific inhibitor or genetic modification. Compounds in the screen that give a phenotypic change under the “synthetic lethal” conditions may be more rapidly associated with their molecular targets if genetic analyses of the compensatory pathways are carried out in parallel [40]. This was exemplified for the discovery of inhibitors of glucose transport from a DOS library [41]. The switch between oxidative phosphorylation to glycolysis as the primary metabolic source of ATP for cell energy requirements is a long-recognized phenomenon of cancer cells (the Warburg effect) and of major interest for anti-cancer drug discovery [42]. A 955-member DOS library of stereochemically pure monocyclic and fused pyrrolidinones was assembled from tandem intermolecular Michael addition—intramolecular amidation starting from β-keto esters and primary amines (Scheme 8). The pyrrolidinone core was selected based on its widespread occurrence in bioactive natural products and drugs. The library was screened in highly glycolytic A549 human lung cancer cells in the presence of the cytochrome c reductase inhibitor, antimycin D which suppresses oxidative phosphorylation and sensitizes the cells to inhibitors of glucose metabolism or glucose uptake. Two compounds **21** and **22** were identified that inhibited ATP production in the presence of antimycin D but not when given as single agents. By profiling the compounds in a panel of cancer and non-tumorigenic cell lines with different dependencies on glycolysis *versus* oxidative phosphorylation, it was observed that the compounds gave a similar pattern of activity to known inhibitors of glucose uptake. Follow up studies demonstrated that **21** and **22** were inhibitors of the glucose transporter GLUT1 (K_i_ 1.2 and 0.8 µM, respectively).

**Figure 6:**
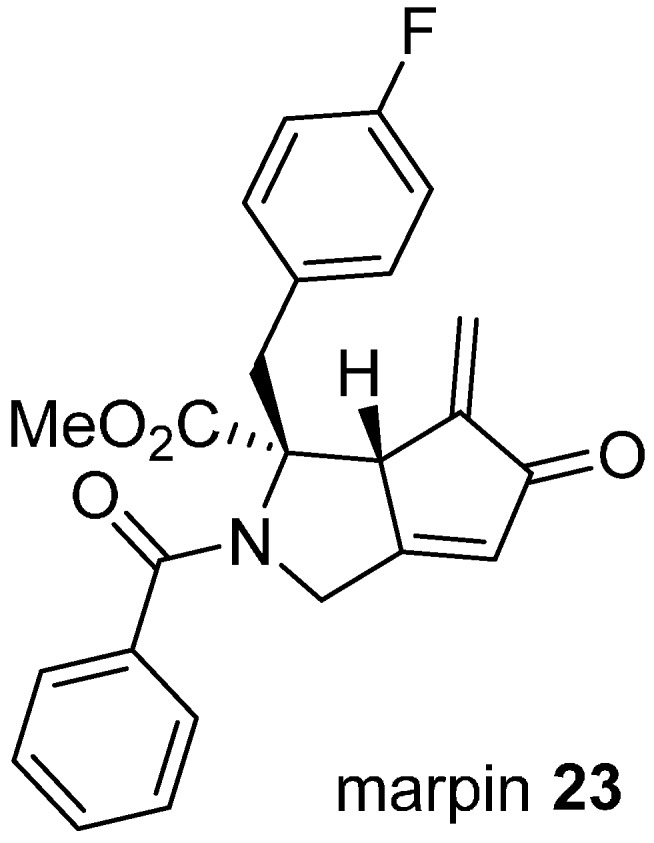
Structure of an inhibitor of DNA-damage response protein activation.

Phenotypic screens can be defined to target modulating ligands to specific pathways, as illustrated in the discovery of marpin (**23**; Figure 6) from a cell-based assay for inhibitors of hydroxyurea-induced phosphorylation of the DNA damage response protein, checkpoint kinase 1 CHK1 [43]. CHK1 is phosphorylated and activated in response to DNA damage, resulting in cell cycle arrest and damage repair, providing an intrinsic resistance mechanism to conventional DNA-targeted chemotherapy. Marpin (IC_50_ 7.7 µM) originated in a library of skeletally diverse cyclic scaffolds prepared from the carbocylization and cyclocarbonylation reactions of allene-ynes and ene-allenes. The compound sensitized p53-deficient cells to DNA-damaging agents but was not an inhibitor of ATR, the upstream activator of CHK1. The mechanism of action is possibly covalent through Michael addition reactions since reduction of the exocyclic alkene to a pendant methyl group reduced activity by more than ten-fold. To identify the molecular target(s), an affinity resin based on marpin was used to pull down candidate proteins, although the identities of these have not yet been disclosed.

**Scheme 9:**
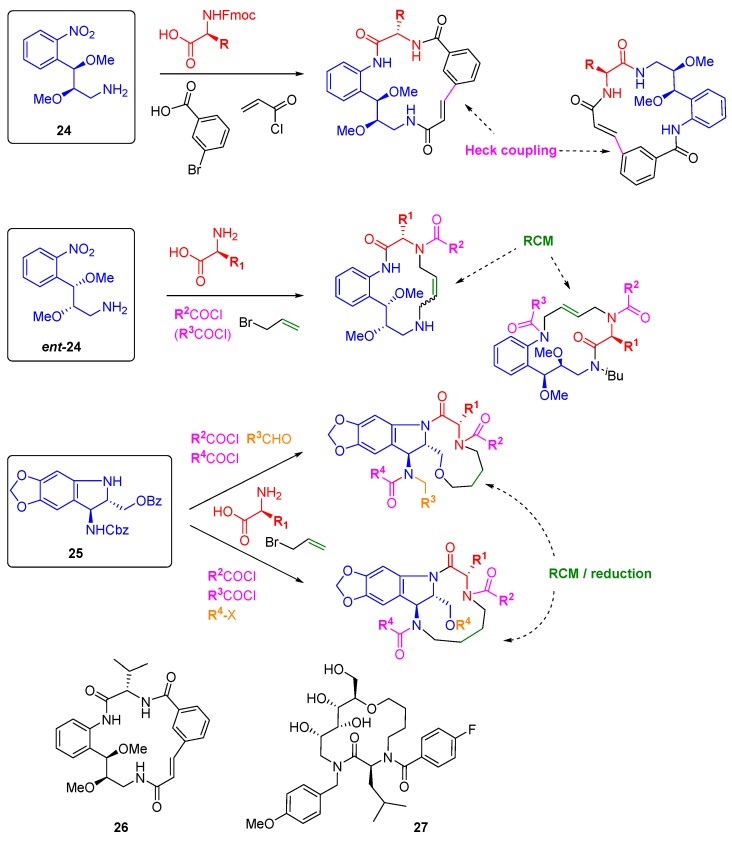
DOS macrocyclic libraries and the structures of macrocyclic angiogenesis inhibitors.

Angiogenesis is a hallmark behaviour of cancer that can be easily interrogated through phenotypic screens in zebrafish embryos. The groups of Arya and Kitambi have made a systematic study of the preparation of DOS libraries of macrocycles and their assessment as anti-angiogenic agents [44,45,46,47]. The choice of macrocyclic scaffolds was driven by their ubiquity in bioactive natural products, including those able to modulate protein-protein interactions, and by the relative paucity of this scaffold type in medicinal chemistry approaches [48]. The bi-functional building blocks **24** and *ent-***24** (Scheme 9), accessed through Sharpless asymmetric epoxidation, were elaborated by amide couplings and macrocyclised either through intramolecular Heck coupling or by RCM to give 17- or 14-member rings, respectively [45,46]. Varying the order of introduction of the building blocks allowed for scaffold variation in each case, and replacement of the alkylamine or nitro groups of **24** by hydroxyl groups gave further diversity. A similar amide coupling and RCM strategy was applied to stereochemically diverse carbohydrate-derived stereo-tetrads to prepare 14-membered glycohybrid macrocycles, e.g., **27** [44]. Ring closing metathesis was also the key step in the construction of a 39-membered library of macrocycles based on the tri-functional aminoindoline **25** [47]. Here, the additional substitution points on the core allowed for the formation of fused or bridged macrocycles. A number of compounds were identified as inhibitors of angiogenesis and/or cell motility during early development of zebrafish embryos, including **26** and **27** which completely inhibited angiogenesis at 2.5 µM concentrations, although the molecular targets have not yet been reported.

**Scheme 10:**
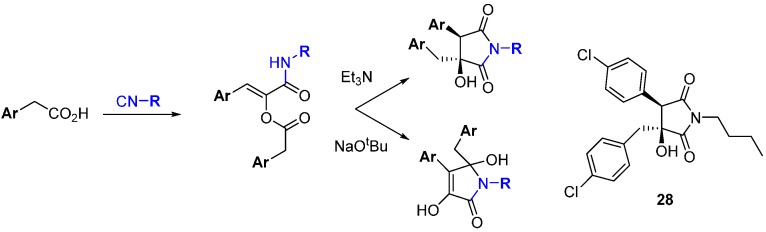
A multi-component reaction and divergent rearrangement leads to inhibitors of angiogenesis.

A novel base-mediated rearrangement of α-acyloxyacrylamides produced in a Passerini-like multi-component reaction was used to prepare a DOS library of five-membered heterocycles from which angiogenesis inhibitors were identified (Scheme 10) [49]. Starting from three isocyanides and three arylacetic acids, a set of 27 compounds was made where production of two scaffolds was controlled by the strength of the base used. Triethylamine gave the product of migration of the *O*-acyl group to nitrogen, followed by intramolecular aldol condensation. In contrast, the stronger alkoxide base gave the product of enolate attack on the amide after the initial migration. Compound **28** inhibited angiogenesis in human endothelial progenitor cells (IC_50_ 5.2 µM).

**Figure 7:**
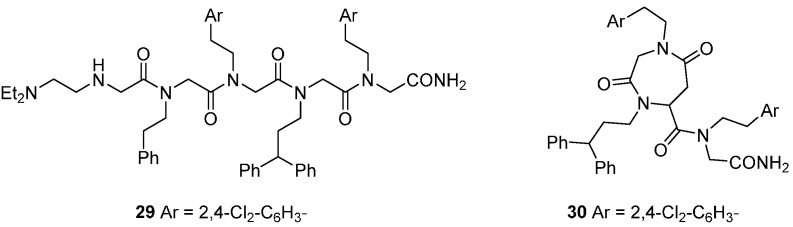
Peptoid inhibitors of the mitochondrial apoptosis complex protein Apaf-1.

DOS concepts have been applied to prepare libraries around scaffold types inspired by the regular repeating motifs of natural biopolymers, specifically peptides and polyketides [50,51,52]. These scaffolds may offer peptidomimetic approaches to the inhibition of protein-protein interactions, such as those involved in the regulation of apoptotic cell death. A set of 5120 peptoid oligomers of N-alkyl glycines generated inhibitors of the apoptosome-mediated activation of procaspase 9, detected in an *in vitro* assay that recapitulated the procaspase-cleavage activity of the complex formed by the proteins rApaf-1, cytochrome c and procaspase-9 with dATP [51]. While the initial trimeric hits were highly lipophilic, modification with terminal polar substituents produced more aqueous soluble analogues, e.g., **29** (IC_50_ *ca.* 10 µM; Figure 7). A fluorescence-labelled derivative of **29** was used to show that the peptoid bound tightly to rApaf-1 *in vitro* (K_d_ 57 nM), but not to other proteins in the complex, and in a non-competitive manner with cytochrome c. To investigate the effects of the peptoid in cells, cell-penetrating derivatives such as the cyclo-peptoid **30** were necessary, since the intrinsic membrane permeability of **29** was low. Compound **30** selectively inhibited mitochondria-mediated apoptosis in several human cancer cell models at 5–10 µM concentrations. Chiral oligomers of pentenoic amides (COPA) that bridge peptoid and polyketide structures are another conformationally constrained scaffold for DOS from which compounds binding to the p53 DNA binding domain *in vitro* were identified [52].

**Figure 8:**
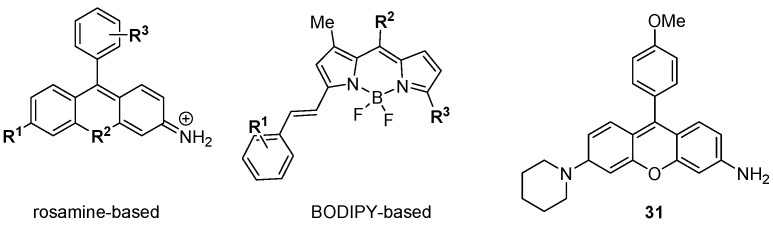
Diversity oriented fluorescence library approach (DOFLA) for differentiating between cancer cell lines from different tissues of origin.

The foregoing discussion has concentrated on the development of small molecule, targeted modulators of biological activity relevant to cancer, but DOS may also be applied to make other functional materials for biomedical research. Chang and colleagues have described a diversity oriented fluorescence library approach (DOFLA) in which a set of 557 molecules containing the rosamine and BODIPY fluorophores was screened against the National Cancer Institute’s panel of 60 human cancer cell lines (NCI 60) using automated fluorescence microscopy [53,54]. Data on the fluorescence phenotypes of the treated cancer cells was collected and clustered according to the change in fluorescence intensity over time. Although the fluorescence probes had limited core scaffold diversity (Figure 8) [55,56] their fluorescent phenotypes were diverse, and the clustered data from 37 probes differentiated between the nine tissues of origin of the 60 cell lines with 98% accuracy. In some cases the probes were highly selective, e.g., **31** gave strong fluorescence in only one of the 60 cell lines (KM12 colon cancer cells).

## 3. DOS Applied to Cancer Drug Discovery Screening Strategies

In the years since the popularisation of DOS, the utility of the approach to probe biological space has been established and increasingly the emphasis has been towards more direct applications in drug discovery including, but not limited to, cancer research. [3,9,10,11,12,13,14,15]. In the following sections we show how DOS strategies have evolved as they are applied to specific medicinal chemistry challenges, from screening efforts to identify bioactive scaffolds, through the incorporation of privileged structure information and ultimately to highly targeted drug discovery.

A number of groups have identified biologically active compounds from DOS libraries using simple phenotypic endpoints such as cancer cell cytotoxicity or inhibition of cancer cell migration. The lack of knowledge of the molecular target often precludes further work on such compounds, but it is nonetheless a first step in demonstrating bio-compatibility for scaffold types not previously or widely explored, and the utility of the DOS transformations that make them. Three-component reactions (3-CR) have proved popular in preparing DOS libraries, such as the one-pot acid-catalysed 3-CR between a range of aldehydes, amines and alkyl/aryl phosphites leading to cytotoxic α-aminophosphonates [57], or novel cytotoxic 1,4-thiazepan-3-ones also made using a 3-CR [58]. The click-chemistry cycloaddition of azides and alkynes is a synthetic transformation that produces triazole scaffolds with physicochemical properties consistent with cellular activity, as shown by antiproliferative carbohydrate-cyclopamine conjugates [59] and cytotoxic 14, 15 and 16-membered macrocylic glyco-conjugates [60].

**Scheme 11:**
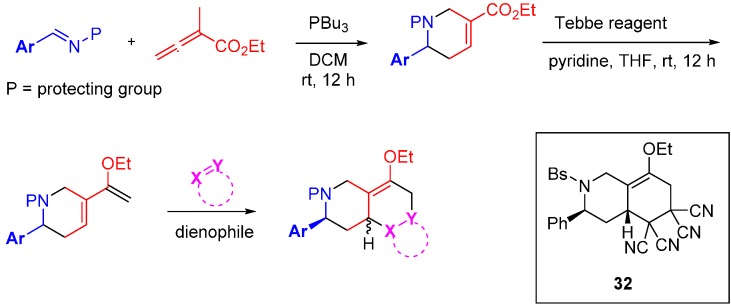
Branched reaction pathway to a DOS library of bicyclic and tricyclic compounds.

The research groups of Fenteany and Kwon elegantly demonstrated the potential of the original DOS approach with a succinct synthesis of 91 heterocyclic structures containing 16 unique molecular architectures [61]. The route incorporated the three tenets of DOS: scaffold; stereochemical; and substituent diversity (Scheme 11). A high yielding phosphine-catalysed cycloaddition was used with electron-deficient allenoates to generate either pyrrolines or tetrahydropyridines, depending on the allenoate substituent pattern. Subjection of the products to a Tebbe reaction installed diene functionalities in good yield. The diene was then intercepted with a diverse range of dienophiles by Diels-Alder reaction in generally modest to good yields, to generate a collection of 16 unique chemical scaffolds. It was found that the pyrrolines underwent cycloaddition to generate a single diastereomeric product while the tetrahydropyridines yielded a range of diastereoselectivities (up to 10:1). The library was screened for the inhibition of cell migration, one of the hallmark behaviours of invasive solid tumours, using MDA-MB-231 human breast cancer cells. Three of the sixteen scaffolds weakly inhibited cell migration in a concentration dependent manner at concentrations below cytotoxic levels (IC_50_ = 15–43 µM). It was also noted that analogue **32** prevented the invasion of MDA-MB-231 cells through the extracellular matrix, albeit at micromolar concentrations.

**Scheme 12:**
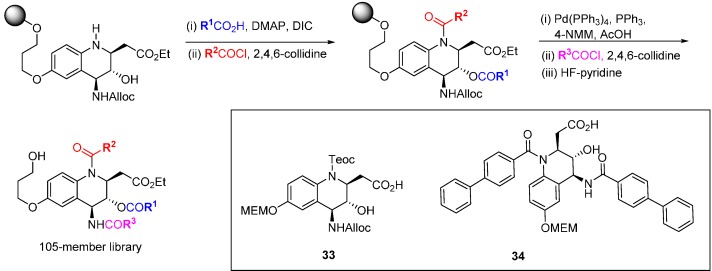
DOS route to a tetrahydroaminoquinoline-derived library.

An alternative to the sometimes high risk approach of screening large collections of compounds from DOS libraries, by which many of the early chemical tool compounds were identified but which has a low hit rate, is to pre-select and concentrate on motifs that are known to possess a degree of bioactivity. When suitable protein target structures are available, virtual screening offers the opportunity to pre-screen potential DOS libraries before synthesis and to guide design, or to focus screening resources on sub-sections of available libraries. This is illustrated in several reports applying DOS to the discovery of new Bcl-family inhibitors. NMR screening of small molecules and fragments to generate SAR and design drug candidates is well established for Bcl-2/Bcl-X_L_ [62], and these techniques have also been applied to evaluate DOS libraries. A substituent-diversity library of 105 compounds was designed around a tetrahydroaminoquinoline core scaffold **33** (Scheme 12) [63]. Using a strategy of orthogonal protection/deprotection the core scaffold was attached to an alkylsilyl macrobead support. Three points of substituent diversity (quinolinamine, amino and hydroxyl functionality) were reacted sequentially with various acylating groups and then cleaved from the resin with HF-pyridine. The 105-member library was analysed by *in silico* screening against both the Bcl-X_L_ and Bcl-2 protein structures, but confirmation of virtual hits by NMR screening with the Bcl-X_L_ protein was not possible due to solubility issues. Removal of protecting groups from the original tetrahydroaminoquinolines gave moderately soluble scaffolds for NMR screening that possessed very weak affinity (K_d_ 0.2–10 mM) for both the Bcl-X_L_ and Mcl-1 proteins. Another round of focussed diversity substitution gave a further nine structures; in particular the biphenyl derivative **34** was found to bind weakly to Bcl-X_L_ (K_d_ 70 µM and Mcl-1 (K_d_ 25 µM). The interaction with the relatively unexplored Mcl-1 target was validated using ^15^N-^1^H HSQC NMR spectroscopy.

The research groups of Bifulco and Basso have also investigated the interactions of a DOS library with the anti-apoptotic protein Bcl-X_L_ [64]. A virtual library of compounds possessing skeletal, stereochemical and substituent diversity was generated from a pluripotent oxabicylic β-amino acid that underwent an Ugi multicomponent reaction with a range of aldehydes, isocyanides and alcohols. Scaffold diversity generating Diels-Alder, ring-opening metathesis (ROM) or RCM reactions were enumerated virtually, and further substitution of the structures was explored, for example using Suzuki couplings. The virtual library generated possessed seven distinct architectures and these compounds were interrogated for potential bioactivities using *in silico* screening against the BH3 binding groove of Bcl-X_L_ and through a NMR-based binding assay of selected compounds. Information from the virtual and NMR screens informed the design of a second round of compounds, but competitive displacement assays with the fluorescent derivative of the native Bak peptide (K_d_ 120 nM) did not show appreciable displacement with any of the compounds prepared.

**Scheme 13:**
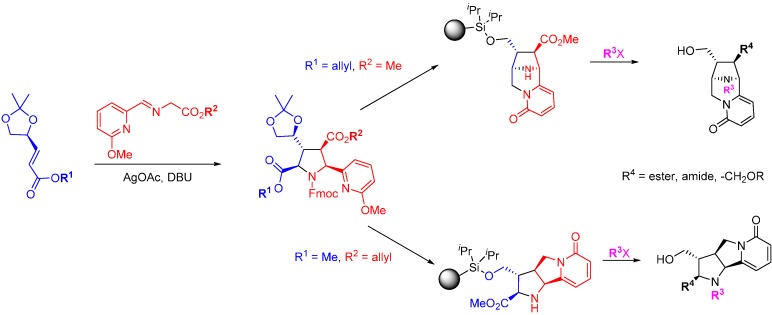
Synthetic route to a cytisine-inspired pyridone library.

In contrast to the two preceding focused DOS library approaches, Infinity Pharmaceuticals adopted a large screening library programme and applied this to Bcl-2 and Bcl-xL. Cytisine, a nicotinic receptor antagonist and phosphatase inhibitor was used as the starting point for natural product inspired DOS and a 15,000-member library was prepared (Scheme 13) [65]. A Horner-Wadsworth-Emmons reaction gave α,β-unsaturated esters followed by a [3 + 2] cycloaddition of glycine-derived azomethine ylides to yield the key cycloadducts. These were converted into two core scaffolds (and their enantiomers) using identical protocols of palladium-catalysed allyl deprotection and ester reduction. The free alcohols were converted to mesylates and cyclisation of the pyridine nitrogen afforded the new ring junction. Removal of the acetal protecting groups and oxidative cleavage of the resulting diols gave the free primary alcohols for attachment of the core scaffold to silicon-functionalised Lanterns (an encapsulated solid support) [66]. While on the Lanterns the methyl esters were converted into the main library of amides, acids and alkyl ethers, and the pyrrolidine nitrogens were alkylated, with the final step being fluoride-mediated cleavage of the library products from the resin. The library was screened at 10 mM for binding affinity against Bcl-2 and Bcl-xL using a fluorinated-BH3 peptide displacement assay. The hit rate for the library was 1.1% for Bcl-2 and 0.2% for Bcl-xL. Several compounds exhibited selectivity for Bcl-2 over Bcl-xL with the most potent example *ca.* K_d_ 1 µM.

**Scheme 14:**
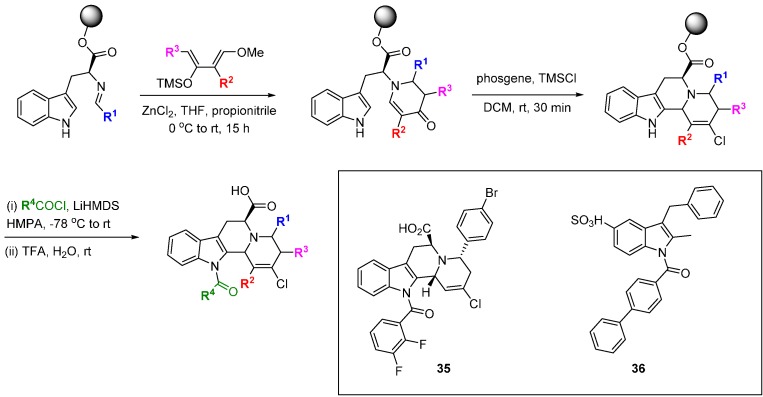
Synthesis of a natural product inspired library of protein phosphatase inhibitors.

Waldmann’s group have applied the BIOS approach to prepare four distinct collections of compounds based on natural products and medium-sized rings, which were assayed for protein phosphatase inhibition [67]. The natural product inspiration was assessed against the criteria of structural classification of natural products (SCONP) as a pre-validation of the library plan. In total 2383 compounds were screened for phosphatase inhibition and details for one of the four scaffolds are shown in Scheme 14. Indoloquinolizidines were prepared by the reaction of tryptophan-derived imines with a range of aldehydes, followed by Lewis acid-mediated, tandem Mannich-Michael reaction with electron-rich dienes in modest diastereoselectivity, and a Pictet-Spengler-type reaction to generate the core scaffold. Finally, the indole was functionalised by *N*-acylation and the products were cleaved from the solid support with acid. The indoloquinolizidine library was based on the yohimbane alkaloids which showed phosphatase inhibition (22–64 µM inhibitors of Cdc25A). The 450 diastereomerically pure library components contained two weak inhibitors of Cdc25A but most interestingly, 11 compounds showed <10 µM inhibition of the phosphatase MptpB (e.g., **35**; IC_50_ 1.1 µM), and 9 of the 11 compounds did not inhibit any other phosphatases at concentrations up to 100 µM. These are the first reported inhibitors of MptpB and structural simplification to tricyclic and bicyclic indole derivatives identified nanomolar inhibitors, such as **36** (IC_50_ 400 nM).

**Scheme 15:**
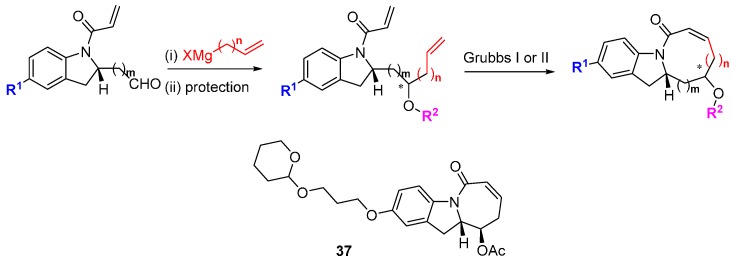
DOS route to tricyclic indolines.

Also taking a privileged scaffold approach based on indoline alkaloids, Arya and co-workers designed a skeletally diverse DOS screening library that provided inhibitors of focal adhesion kinase (FAK), a protein involved in tumorigenesis and metastasis [68]. *N*-Alloc-protected 2-formylindolines or homologues were functionalised by addition of allylic or homoallylic Grignard reagents under Felkin-Anh control (Scheme 15). After alcohol protection, the resulting proximal vinyl groups were reacted by RCM to afford 6-, 7- and 8-membered rings fused to the indoline scaffold. Compound **37** inhibited FAK activity (30% inhibition at 30 µM) but its mode of action (ATP-competitive or allosteric) was not determined, although docking studies suggested an ATP-site inhibitor. In a cell proliferation assay **37** had a cytotoxicity of 39 µM and reduced expression of FAK autophosphorylation on pY397. The inhibitor also reduced MDA231-M cell invasion and motility, which may at least in part be due to FAK inhibition.

**Scheme 16:**
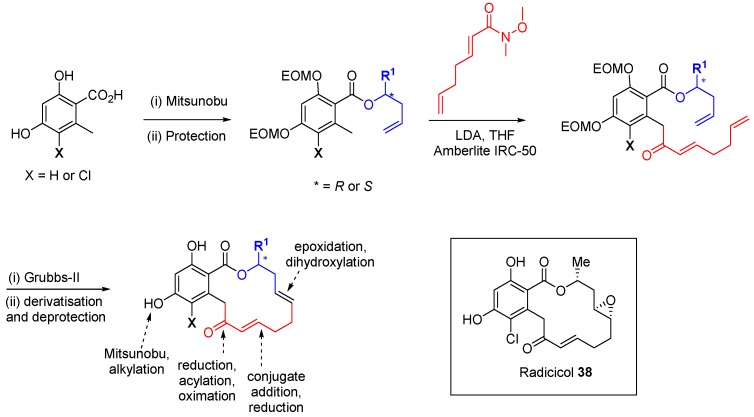
Synthetic route to pochonin-inspired macrocyclic kinase inhibitors.

The Winssinger group has identified kinase activity within a DOS library of pochonin analogues [69]. The pochonin class of natural products is exemplified by radicicol (**38**; Scheme 16) a 20 nM ATP-competitive inhibitor of the chaperone HSP90. Other members of the family are inhibitors of protein kinases, despite the significant differences in protein fold and cofactor conformation between kinase and HSP90 ATP-binding pockets. The core macrocycle was appended with five points of diversity. The synthesis involved an optional chlorination at the 5-position of the resorcinol acid starting material and a solid-supported Mitsunobu reaction to esterify the acid group with enantiomerically pure homoallylic alcohols. Protection of the two phenolic groups with the EOM group allowed deprotonation of the tolyl group with LDA and reaction of the anion with an α,β-unsaturated Weinreb amide. The final ring-closing step was performed using Grubbs II-mediated RCM. Further derivatisation of the macrocycles followed, including ketone reduction and acylation of the resulting alcohol to form dienes, conjugate addition to the α,β-unsaturated ketone, and reduction, epoxidation or dihydroxylation of the non-conjugated double bonds. Removal of the EOM protecting groups allowed the option of phenolic *O*-derivatisation. In total 113 macrocycles were prepared and 84 compounds were screened at 10 µM against a panel of 24 kinases. A hit rate of 14% was found with 12 compounds demonstrating >50% inhibition of at least one enzyme, of which 8 showed modest inhibition on titration (IC_50_ 8–50 µM). Further developments of resorcyclic macrolactone scaffolds preserving the reactive enone functionality have led to a number of irreversible kinase inhibitors that target specific cysteine residues, including VEGFR kinase inhibitors with *in vivo* efficacy [70].

**Scheme 17:**
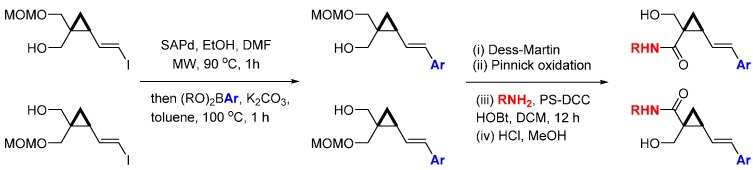
Synthetic route to a cyclopropane-containing library of potential kinase inhibitors

A ligand-free Suzuki coupling reaction was developed for the synthesis of conformationally restricted cyclopropanes with three-dimensional diversity [71]. Combining the concepts of DOS and fragment-based drug design (FBDD) led to library of 90 compounds that were screened against a panel of 20 kinases (Scheme 17). The rigid geometry of the cyclopropanes allowed the placement of aryl fragments, chosen as potential kinase hinge-binding motifs, in distinct geometrical locations, giving chiral products enriched in sp^3^-carbons, an often under-represented feature for screening libraries as discussed in the introduction. The enantiomeric cyclopropane vinyl iodides were reacted with a range of aryl boronic acids followed by oxidation of the free alcohol to the carboxylic acid using sequential Dess-Martin and Pinnick reactions. A second round of substituent diversity was achieved through amide formations using solid-supported DCC, followed by MOM deprotection. The kinase screening panel results indicated that the regiochemistry of the cyclopropane substituents determined the kinase inhibitory activities, with modest inhibition of FLT3, JAK3, PDGFRα, and TRKA (20%–50% inhibition at 10 µM) observed, for example.

**Scheme 18:**
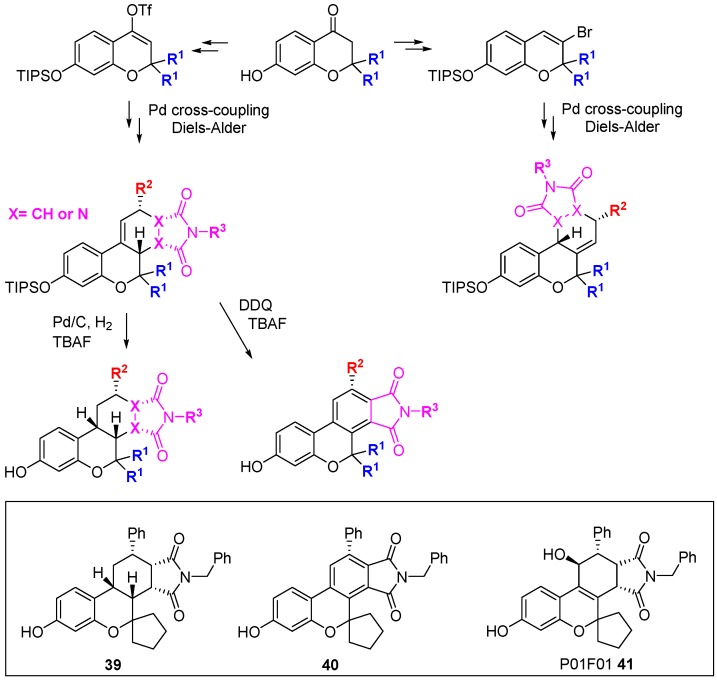
DOS of privileged benzopyran containing polycycles and identification of an androgen receptor antagonist.

Park and co-workers have developed a branching DOS library inspired by natural products containing the privileged benzopyran ring system [72,73]. The DOS strategy involved balancing two seeming opposites: biased incorporation of privileged structures and unbiased diversity. In total, 22 unique core structures were prepared with limited appendage diversity; this enabled the side-by-side evaluation of core structural diversity. From a 3- or 4-(*pseudo*)halo substituted benzopyran, three key reactions were performed to generate the diastereo-enriched library; Diels-Alder cycloaddition, click chemistry, and palladium-catalysed cross-coupling. In particular, Stille cross-coupling followed by *endo*-favoured Diels-Alder cycloaddition generated steroid-like tetracycles (Scheme 18). U-shaped structures were generated by hydrogenation while DDQ-mediated oxidation gave flat aromatic systems. Comparison of examples **39** and **40** highlighted the power of three-dimensional architectures to provide differential biological activity: A viability assay in A549 lung carcinoma cells demonstrated the cytotoxicity of **39** (1.0 µM) *versus* **40** (63.9 µM). Using a cell-based reporter assay in LNCaP human prostate adenocarcinoma cells, screening of a 2000-member benzopyran DOS library, followed by focussed screening of 19 second-round derivatives identified P01F01 (**41**) as an antagonist of the androgen receptor [73,74]. The compound was also shown to inhibit transcription mediated by androgen receptor mutants that are resistant to inhibition with conventional antagonists such as bicalutimide.

Pappo has described a DOS-type approach to coumestrol-based selective oestrogen receptor (ER) modulators [75]. A FeCl_3_/O_2_ oxidative coupling reaction between β-keto esters and phenols was used to prepare a range of benzofurans, followed by deprotection and lactonisation to afford the coumestans. Initial *in silico* studies suggested a binding mode in the ER active site. This was confirmed biologically by screening of the compound collection against the breast cancer cell lines, MCF-7 (oestrogen dependent) and MDA-MB-321 (oestrogen independent). The most potent compound prepared inhibited MCF-7 cell proliferation (IC_50_ 9 nM) and initial SARs were identified.

## 4. DOS Applied to Specific Molecular Starting Points in Cancer Drug Discovery

**Scheme 19:**
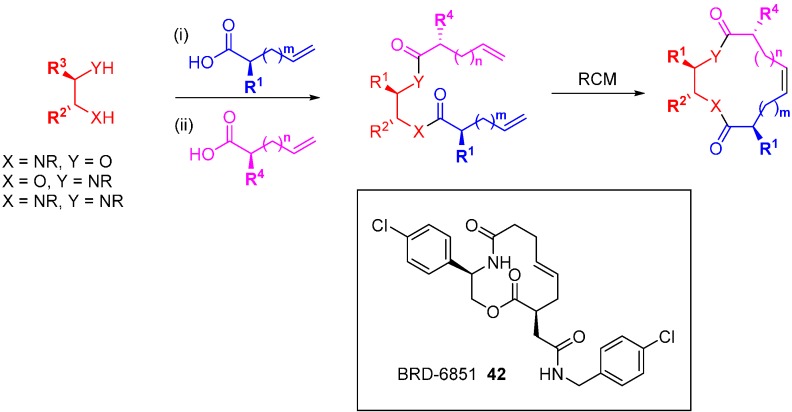
DOS route to robotnikinin-derived analogues and the structure of the Smo antagonist BRD-6851 (**42**).

DOS has been applied in drug discovery to populate a chemical library with diverse modifications starting from specific molecules with known bioactivity. The macrocyclic Sonic Hedgehog (Shh) signalling pathway inhibitor, robotnikinin (**11**; Figure 5) was discovered from a screening campaign of a DOS library, as discussed above. A range of diverse robotnikinin analogues were prepared via a *build-couple-pair* strategy (Scheme 19) [76]. Interestingly, this led to a series of compounds that inhibited Shh signalling by an alternative mechanism to robotnikinin. Using a protecting group strategy various alkenoic groups were attached to either amino alcohols or diamines, with variations to both the linker lengths and side chains, and the synthesis of the macrocycles was completed by RCM. SARs were established from inhibition studies using a Shh-induced C3H10T1/2 alkaline phosphatase assay compared to the known inhibitor cyclopamine (IC_50_ 600 nM). Robotnikinin (**11**) was weakly active (IC_50_ > 25 µM) but reversing the positions of the macrocycle oxygen and nitrogen led to a more potent inhibitor (IC_50_ 5 µM). A systematic exploration of simple structural changes led to the following observations: methylation of the macrocyclic nitrogen was tolerated; inversion of the 2-position stereochemistry ablated activity; 11-position substitution was acceptable; the minor product of the RCM, the *Z*-olefin, was more active than the *E*-olefin; the 6-substituent needed to be lipophilic; truncated analogues were inactive suggesting a large pharmacophore; and finally, a 4-chlorophenyl group at the 2-position was most potent, at 400–600 nM depending on the assay format, leading to BRD-6851 (**42**). This compound and others blocked the Shh pathway by inhibiting the GPCR, Smoothened (Smo). Competition studies with the Smo agonist purmorphamine, and retention of activity in cell lines lacking the Shh receptor Patched suggested that **42** was a Smo antagonist, a completely different mode of Shh pathway inhibition from robotnikinin, the starting point of the DOS library.

**Scheme 20:**
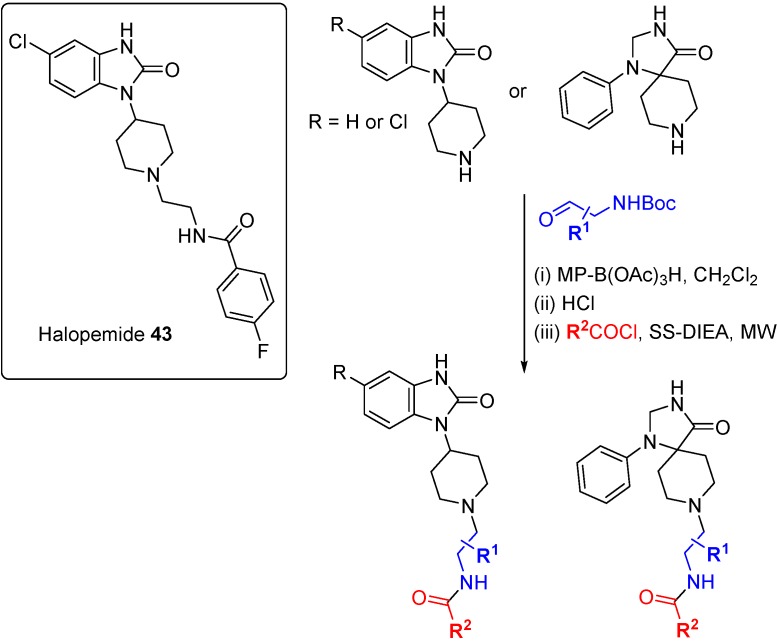
DOS-type exploration of SAR for PLD1/2 inhibitors derived from halopemide.

A DOS approach was used to identify the first isoform-selective phospholipase D (PLD) inhibitors [77]. PLD is responsible for the synthesis of phosphatidic acid, a secondary messenger implicated in both GPCR and tyrosine receptor kinase signal transduction pathways, and inhibition of PLD blocks breast cancer cell metastasis. A previously published inhibitor, the psychotropic compound halopemide (**43**; Scheme 20), was the starting point for a DOS library to develop dual-PLD1 and PLD2, and also isoform selective PLD inhibitors. Using a diversity-oriented approach to explore SAR, halopemide was dissected into modular sections and a 263-member library was prepared using three alternate scaffolds, three linkers and 30 amide caps. The entire library was screened at a single concentration against PLDs and 30 compounds were followed-up based on activity, selectivity and structural diversity. Remarkable isoform specificity for both PLD1 and PLD2 was found *in vitro*, with central scaffold rather than appendage diversity the key driver of selectivity, although (*S*)-methyl groups on the linker conferred PLD1 selectivity to otherwise dual PLD1/2 inhibitors. Cellular activity against PLD isoforms was also demonstrated for several compounds from the DOS campaign but most importantly, the inhibitors were shown to block invasion in breast cancer cell lines (MDA-231, 4T1 and PMT). siRNA knockdown studies confirmed the cell invasion blocking effects were due to either dual PLD1/2 or selective PLD2 inhibition but not selective PLD1 inhibition.

**Scheme 21:**
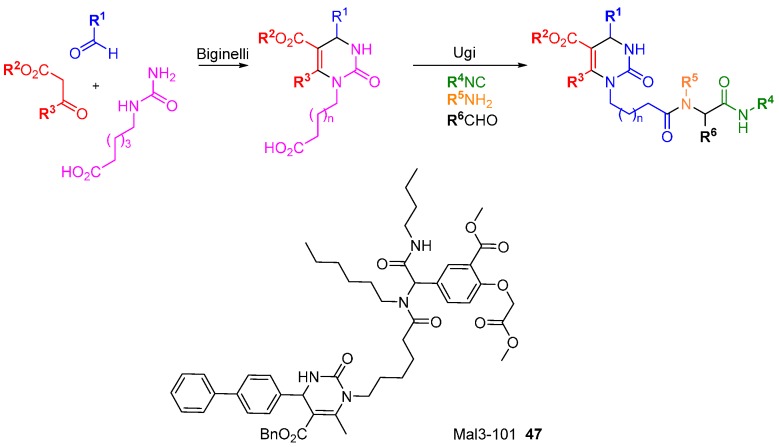
A tandem multi-component reaction route to analogues of the HSP70 inhibitor Mal3-101.

A screen of compounds with similar structures to 15-deoxyspergualin and NSC 630668-R/1 identified amongst others, Mal3-101 (**47**; Scheme 21) as an inhibitor of J-chaperone-stimulated HSP70 ATPase activity [78]. Hsp70 is a molecular chaperone with pro-survival properties in relation to misfolded proteins and is often up-regulated in cancer cells. HSP70 is a validated and promising target in cancer therapy but to date very few modulators have been identified. The synthesis of **47** was accomplished via two MCRs; a Biginelli reaction to prepare the dihydropyrimidinone core, then an Ugi reaction under microwave irradiation to complete the synthesis. Mal3-101 was used as the inspiration for a DOS-like library [43] to prepare near-analogues (all retaining the dihydropyrimidinone core) with improved anti-proliferative activity, anti-malarial activity, and SV40 activity.

**Scheme 22:**
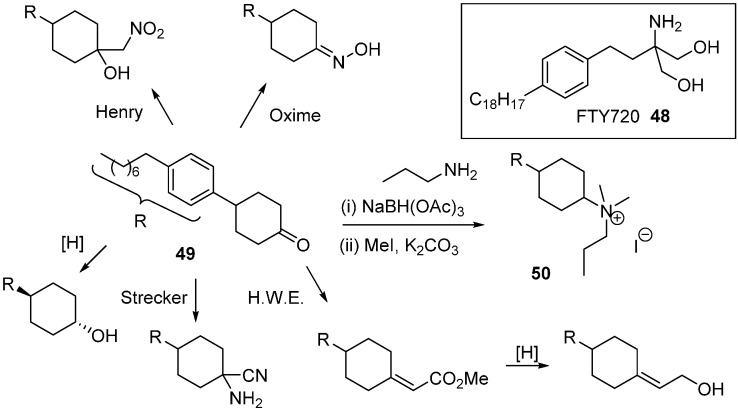
Diversity strategy for head group variation of sphingosine kinase inhibitors.

Sphingosine kinase (SphK) is a cancer target which plays a role in cell survival. The Santos research group was inspired by FTY720 (**48**; Scheme 22), an immunomodulatory drug that is phosphorylated by SphK to generate a sphingosine 1-phosphate mimic [79]. A DOS library was generated where the key diversity point came from the divergent reactivity of the cyclohexanone **49** to generate novel head groups. Amino head groups were identified as hits when the compound collection was screened at 100 µM against SphK1 and SphK2. Following a second round of synthesis focussed on amino variants, quaternary ammonium salts proved to be the most potent (e.g., **50**; *K*_i_ 8 µM) with approximately 3 to 4-fold selectivity for SphK1 over SphK2.

**Scheme 23:**
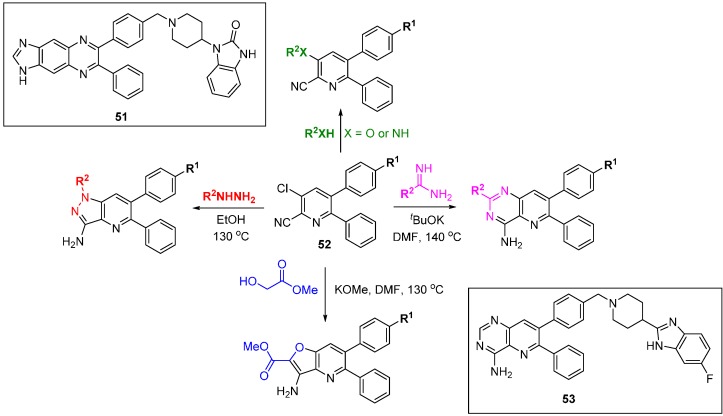
DOS approach to identify alternative scaffolds for allosteric Akt inhibitors.

There have been two reported DOS approaches applied to explore the SAR of allosteric Akt inhibitors [80,81]. The PI3K-Akt signalling pathway is often up-regulated in cancers and regulates diverse functions including cell growth, proliferation, motility and survival. The Merck approach started with the evolved hit quinoxaline **51** (Scheme 23) [80] and involved the assembly of four skeletally diverse pyridine-containing scaffolds from common precursor **52**. The library was screened for Akt1 and Akt2 inhibition and compound **53**, an example of the pyridopyrimidine scaffold, was a potent Akt1 inhibitor *in vitro* and in cells (IC_50_ 18 nM and 227 nM, respectively). Importantly, **53** had a >10-fold selectivity window over the isoforms Akt2 and Akt3, and other AGC-family kinases SGK, PKA, and PKC. In a pharmacokinetic study in dog the compound had a long half-life and low clearance. Researchers at Astra-Zeneca began with the clinical candidate MK-2206 and the DOS approach centred on different ring fusions around a core pyridine [81], ultimately leading to a simpler core scaffold with low clearance and very high free drug concentrations in plasma.

## 5. Conclusions

The examples collected above clearly show that diversity-oriented approaches can deliver useful new chemical tools for cancer biology, and can be productively applied to early drug discovery. While the importance of skeletal, stereochemical and substituent diversity remains fundamental, the field has evolved as DOS strategies have been used in the context of drug discovery. The discovery of chemical probes through screening of large libraries whose components cover areas of previously unexplored chemical space is now well established, and has generated important pharmacological tools for cancer biology. The challenges in this approach include the relatively low probability of finding hits for a given target or phenotype in an unbiased collection, necessitating large library size, and the substantial effort required to fully characterize the molecular target(s) of compounds identified through some phenotypic screens. As illustrated in this article, there are still molecules eliciting interesting phenotypes in cancer cells for which specific molecular targets have not been identified. Small molecule probes or tool compounds are of limited use for further biological research without knowledge of their targeted interactions and their selectivity [82]. However, it is clear that higher content phenotypic screening or a combination of phenotypic and biochemical screening can go a long way to address these problems and provide well-credentialed chemical tools from DOS libraries. An early grand challenge laid down in chemical genetics was to identify a chemical probe for every human gene product [83]. A recent analysis suggested that, for 58 very well evidenced cancer driver genes less than half had sub-micromolar potent small molecule modulators identified, including targets with three-dimensional structures available [84]. Thus despite rapid advances in the past decade there are many potential cancer drug targets for which small molecule modulators are still unknown [85], and this currently barren probe space undoubtedly extends to other disease areas. There is much to be done, and diversity oriented strategies have the potential to make major contributions.

In the context of target-specific medicinal chemistry, various means of defining the chemical space to be explored by DOS-like strategies have proved successful. This is most frequently performed at the level of skeletal diversity. Although this would seem to contradict the original precepts of structural diversity-led synthesis, the creative tension between simultaneously achieving greatest structural diversity, greatest biological relevance and physiological compatibility is leading successfully to new biologically active molecules. Many recent applications therefore describe using privileged structures, inspiration from natural products or concepts of drug-likeness to pre-populate chemical-biological space. Interestingly, as seen with some examples of both the DOS and BIOS approaches, this does not always guarantee the targeted bioactivity of the starting point(s) will be faithfully recapitulated in the expanded libraries. The distinction between DOS and established medicinal chemistry strategies becomes increasingly blurred as greater focus is placed on specific starting points for library design and the number of diversity points decreases, or scaffold diversity gives way to purely substituent variation.

**Figure 9:**
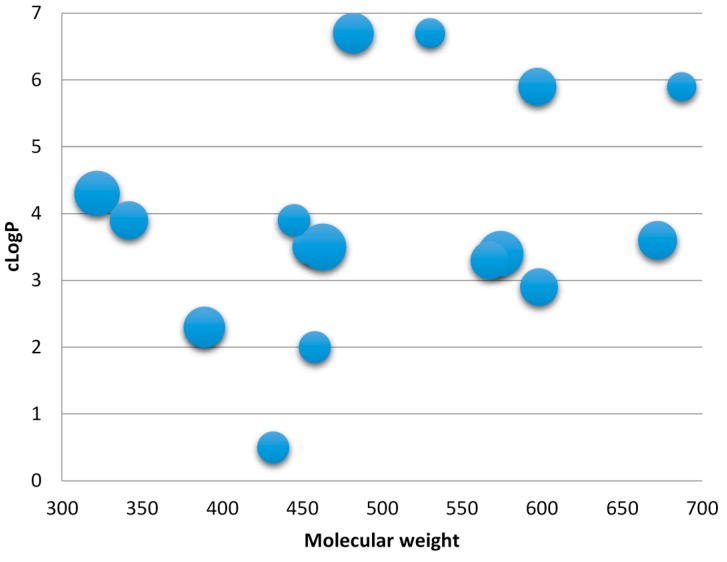
A graph of clogP *vs.* molecular weight for 17 compounds summarised in this article and identified as chemical endpoints for which K_d_, K_i_ or IC_50_ determinations against specific targets in cell-free assays were reported. The size of the circles is proportional to pK_d_ or pIC_50_, where pK_d_ = −log_10_ (K_d_) (range from 4.6 to 7.3).

Importantly, the new chemical space accessible through DOS can provide molecules that interact with less tractable targets or with targets with little pedigree for modulation by small molecules, as evidenced by the identification of protein-protein interaction inhibitors and modulators of transcription factors. For the purposes of drug discovery, access to new chemical space needs to be balanced with the danger of “molecular obesity” inherent in any synthetic strategy that builds up molecular size and lipophilicity, particularly relevant to DOS when substituent variation is the major component of diversity [86]. Analysis of the size and calculated lipophilicities of seventeen of the DOS- and BIOS-derived tool or lead compounds highlighted in this review, for which target-specific K_d_, K_i_ or biochemical IC_50_ from cell-free assays were reported, is informative; with the caveat that this is a limited survey of one disease area and only the most potent compounds in each report where this data was available are included. Limitations aside, this snap-shot (Figure 9) shows half of the compounds occupying physicochemical property space at or above the upper bounds typically associated with drug-likeness (e.g., cLogP > 5, MW > 500). On the other hand, in at least half of these examples of DOS outputs these properties can be reconciled; compound libraries that balance diversity, novelty and control of physicochemical properties are highly desirable [87]. The range of potencies in Figure 9 indicates some molecules would be considered as early hit matter (pK_d_ or pIC_50_ < 6) requiring substantial improvement to generate fully developed probe molecules or useful drug discovery leads, while others are potentially more immediately useful chemical tools [82].

The use of cell-based screening provides an initial assurance that the physicochemical behaviours of molecules identified as hits are compatible with cellular activity. Drug-likeness also concerns the physicochemical property ranges compatible with bioavailability in whole organisms and these considerations often concentrate on the aqueous solubility, lipid permeability and propensity for metabolism or active transport that govern oral bioavailability. These features may be less immediately relevant for chemical tools intended for early cell biology studies, but higher molecular size and lipophilicity are also associated with a greater risk of promiscuous activity [88]. The selectivity of putative chemical tools is one of the most important determinants of the reliability of biological findings derived from their use [82].

Some of the most recent reports show how DOS strategies allow rapid exploration of a large space around single, well defined chemical leads, to enable medicinal chemistry or chemical biology studies. The integration of *in silico* screening against specific targets and virtual library enumeration should be a powerful combination to guide and maximize the contributions of DOS to the discovery of targeted anti-cancer therapeutics. Recent publications suggest that DOS may be applicable in fragment-based drug discovery [71,89,90] and it would be expected that the trend to incorporate DOS with other medicinal chemistry strategies in anti-cancer drug discovery will continue productively.
